# Preliminary Effectiveness of the Comprehensive Oncology Rehabilitation and Exercise (CORE) Clinical Workflow Algorithm on Health Outcomes During Nonmetastatic Breast Cancer Care

**DOI:** 10.1155/ijbc/8894250

**Published:** 2026-06-11

**Authors:** Lea Haverbeck Simon, Carson Saviers-Steiger, Emily R. Dunston, Susan L. Zickmund, Pamela A. Hansen, Cornelia M. Ulrich, Paul C. LaStayo, David Steinberg, Christopher S. Noren, A’Lisha Finch, Leanne Seckinger, Emma Braun, Jonathan Chipman, Sonal Oza, Kirstyn E. Brownson, Adriana M. Coletta

**Affiliations:** ^1^ Cancer Control and Population Sciences Program, Huntsman Cancer Institute, University of Utah, Salt Lake City, Utah, USA, utah.edu; ^2^ College of Dental Medicine, Roseman University of Health Sciences, South Jordan, Utah, USA, roseman.edu; ^3^ Huntsman Cancer Institute, University of Utah, Salt Lake City, Utah, USA, utah.edu; ^4^ Department of Health and Kinesiology, University of Utah, Salt Lake City, Utah, USA, utah.edu; ^5^ Veterans Affairs Salt Lake City Health Care System, Salt Lake City, Utah, USA; ^6^ Division of Epidemiology, Department of Internal Medicine, University of Utah School of Medicine, Salt Lake City, Utah, USA, utah.edu; ^7^ Department of Physical Medicine and Rehabilitation, University of Utah School of Medicine, Salt Lake City, Utah, USA, utah.edu; ^8^ Department of Population Health Sciences, University of Utah, Salt Lake City, Utah, USA, utah.edu; ^9^ Department of Physical Therapy and Athletic Training, University of Utah, Salt Lake City, Utah, USA, utah.edu; ^10^ Therapy Services, University of Utah Health, Salt Lake City, Utah, USA; ^11^ Department of Rehabilitation Medicine, Emory University School of Medicine, Atlanta, Georgia, USA, emory.edu; ^12^ Department of Surgery, University of Utah School of Medicine, Salt Lake City, Utah, USA, utah.edu

## Abstract

**Background:**

The Comprehensive Oncology Rehabilitation and Exercise (CORE) pilot trial aimed to test the feasibility and acceptability of a clinical workflow algorithm that integrated exercise and rehabilitation services from breast cancer diagnosis throughout the first 24 weeks of care. Here, we investigated the preliminary effectiveness of the CORE algorithm compared with standard of care (SOC) on changes in physical function, health‐related quality of life (HRQoL), and exercise engagement in women newly diagnosed with Stage I–III breast cancer with plans for surgery as first‐line treatment.

**Methods:**

Seventy‐two women were randomly assigned in a 2:1 ratio to CORE or SOC. All participants completed study assessments at three time points that aligned with routine breast surgical oncology clinic visits: surgical consultation (i.e., baseline), postoperative, and 24 weeks postoperative. The following outcomes and associated assessments were carried out in the clinic: physical function: PROMIS physical function survey (primary function assessment), five‐time chair stand, 10‐m walk, back scratch, and QuickDASH survey; HRQoL: FACT‐B survey; and exercise engagement: modified Godin physical activity survey (primary engagement assessment) and accelerometer wear for 1 week following each clinic visit.

**Results:**

Fifty‐nine participants had evaluable data, with the majority having Stage I disease (83%), being primarily White (75%) and non‐Hispanic (90%), and having a median age and BMI of 58 years and 26.0 kg/m^2^, respectively. Mean difference in change in PROMIS physical function score from baseline to 24 weeks postoperative between CORE and SOC was not statistically significant (−1.86, 95% CI −6.02 to 2.3). A modest advantage in exercise engagement was observed in the CORE arm (Godin: 5.46, 95% CI −1.06 to 11.98; effect size: 0.36, 95% CI −0.07 to 0.78; accelerometry: median difference 12 min, bootstrapped 95% CI −37 to 40).

**Conclusion:**

The CORE clinical workflow algorithm demonstrates promise in improving exercise engagement. More work is needed in an adequately powered trial to confirm these findings and evaluate effectiveness on other outcomes.

**Trial Registration:**

ClinicalTrials.gov identifier: NCT04594473

## 1. Introduction

Breast cancer remains the most prevalent and second deadliest cancer among women [1]. Despite significant improvements in screening and treatments that have contributed to the prevention of well over 500,000 breast cancer deaths in the United States between 1989 and 2022 [2], the disease and its treatments exert deleterious effects on the lives of those affected. Both breast cancer and its treatments are associated with a wide range of adverse side effects, including significant deficits in physical function [3] and health‐related quality of life (HRQoL) [4, 5]. While HRQoL appears to improve after treatment completion [6], breast cancer treatment may exacerbate declines in both physical function [7] and HRQoL [8] throughout the cancer care continuum. Common breast cancer treatment modalities, including surgery, radiotherapy, or chemotherapy, have the potential to negatively affect physical function through localized symptoms such as upper extremity swelling and pain with loss of mobility, as well as systemic issues like fatigue and psychological distress [9]. The maintenance of physical function is crucial as it independently reduces the risk of all‐cause mortality in individuals living with cancer [10, 11], and declines in physical function are associated with worse survival outcomes in breast cancer [12].

Exercise has emerged as a powerful therapeutic tool to improve cancer‐ and treatment‐related adverse side effects, especially when implemented across the cancer care continuum from diagnosis throughout survivorship [13–18]. For instance, in women with Stage 0–III breast cancer within 2 months to 5 years of treatment, a prescribed exercise program executed independently improved both self‐reported and objectively measured physical function [19]. Exercise in women with breast cancer during and after treatment also improves physical function and fatigue [20]. Importantly, engagement in exercise after breast cancer diagnosis is linked to a 37% reduction in breast cancer–specific mortality rate [21] and improved breast cancer survival [22], underscoring the critical need to promote exercise engagement throughout the cancer care continuum (i.e., after diagnosis, before, during, and after cancer treatment) in this population.

Both the American College of Sports Medicine (ACSM) and the American Society of Clinical Oncology (ASCO) recommend physical activity (PA) throughout the cancer care continuum [17, 23, 24]. It is recommended that individuals with cancer undergoing active treatment complete at least 90 min of moderate‐intensity aerobic exercise per week [17]. Before and after treatment, 150–300 min of moderate‐intensity aerobic exercise per week is recommended [17]. Regardless of where an individual is on the cancer care continuum, strength training should be completed 2 days per week. However, despite these guidelines and growing evidence from large international trials [25], exercise oncology services are not yet part of routine cancer care.

As such, our team at the Huntsman Cancer Institute (HCI) at the University of Utah developed the Comprehensive Oncology Rehabilitation and Exercise (CORE) clinical workflow algorithm. This algorithmic triaging tool was designed to connect newly diagnosed Stage I–III patients with breast cancer undergoing surgery as first‐line treatment to appropriate exercise and rehabilitation services at the right time. In the CORE pilot trial, these patients were referred to exercise or rehabilitation services before, after, and 6 months after their breast cancer surgery, with the ultimate goal of promoting the transition to independent exercise self‐management. The CORE clinical workflow algorithm was found to be feasible and acceptable among individuals with breast cancer and oncologic clinic staff [26]; however, it is unknown whether its application is associated with improvements in physical function, HRQoL, and exercise engagement throughout the breast cancer care journey. Therefore, the purpose of this investigation was to evaluate the preliminary effectiveness of the CORE clinical workflow algorithm on physical function, HRQoL, and exercise engagement among newly diagnosed nonmetastatic breast cancer patients requiring surgery as first‐line treatment throughout the first 24 weeks of routine breast cancer care. We hypothesized that the CORE clinical workflow algorithm would induce favorable changes in subjective and objective measures of physical function, HRQoL, and exercise engagement compared with receiving 24 weeks of routine breast cancer care (standard of care [SOC]).

## 2. Methods

### 2.1. Study Design and Participants

This investigation aimed to assess the preliminary effectiveness of the CORE clinical workflow algorithm on physical function, HRQoL, and exercise engagement during the first 24 weeks of clinical care in women with nonmetastatic breast cancer as part of the CORE trial. The CORE trial was a 24‐week, mixed‐methods, parallel, randomized controlled pilot trial at the HCI at the University of Utah that tested the feasibility and acceptability of the HCI‐developed CORE clinical workflow algorithm. It was approved by the University of Utah Institutional Review Board (IRB #00137018). The detailed methodology of the CORE trial has been described elsewhere [26], and the methodology description of the present investigation partly reproduces their wording.

Briefly, the CORE trial randomized 72 women with breast cancer to the CORE intervention or SOC in a 2:1 ratio. Women were eligible for this trial if they had nonmetastatic breast cancer (Stage I–III) with plans to undergo surgery as the first treatment modality. Enrolled participants were block randomized based on adherence (yes/no) to national PA guidelines [26]. National PA guideline adherence was met if patients engaged in at least 150 min of moderate‐intensity aerobic exercise or 75 min of strenuous‐intensity aerobic exercise (or any combination thereof), along with strength training twice weekly [27]. Participants randomized to SOC were instructed to maintain their current lifestyle activities throughout the trial period while receiving routine breast cancer care. Participants randomized to the CORE intervention completed the CORE algorithmic triaging tool, administered by clinic staff at the check‐in desk via iPads, which included two short questionnaires: (1) the Patient‐Reported Outcomes Measurement Information System (PROMIS) Physical Function Short Form 8b to assess physical function and (2) the modified Godin‐Shephard Leisure‐Time Physical Activity Questionnaire (Godin) to assess current PA levels. Responses to these questionnaires informed triage to one of three pathways within the algorithm: exercise service, rehabilitation service, or exercise self‐management (i.e., keep exercising as you usually do). The triage was conducted at the three routine clinic visits with breast surgical oncology: (1) initial surgical consultation, (2) postoperative visit, and (3) 24‐week postoperative visit [26].

### 2.2. Outcome Measures

To assess the preliminary effectiveness of the CORE clinical workflow algorithm on physical function, HRQoL, and exercise engagement, several subjective and objective measures were performed in the clinic during the aforementioned routine clinic visits with breast surgical oncology.

### 2.3. Physical Function Assessments

The following assessments of physical function were carried out in the clinic: PROMIS Physical Function Short Form 8b questionnaire, Quick Disabilities of the Arm, Shoulder, and Hand (QuickDASH) questionnaire, five‐time chair stand test, 10‐m walk test, and back scratch test.

#### 2.3.1. PROMIS Physical Function

All participants completed the PROMIS Physical Function Short Form 8b questionnaire [28]. This questionnaire consists of eight questions rated on a 5‐point Likert scale inquiring about activities of daily living (e.g., carrying groceries and vacuuming). Higher scores indicate better physical function.

#### 2.3.2. QuickDASH

The QuickDASH questionnaire consists of 11 questions rated on a 5‐point Likert scale inquiring about the ability to use upper extremities in activities of daily living such as household chores or using a knife to cut food [29], pertinent to individuals with breast cancer who may experience upper extremity complications after surgery. Higher scores indicate greater upper extremity disability.

#### 2.3.3. Five‐Time Chair Stand Test

The five‐time chair stand test assesses the time it takes to rise from a chair, with arms crossed over the chest, for five consecutive times as fast as possible [30]. Participants were instructed to sit in a chair with bent legs at 90°, and the researchers recorded the time it took (in seconds) to complete five chair stands [31].

#### 2.3.4. Ten‐Meter Walk Test

Participants were instructed to walk in a straight, 10‐m line at 0% incline as fast as possible; time to walk the 10‐m course was recorded in seconds to determine gait speed [30]. The walking area was premeasured with rope attached to two small cones at each end. This enabled flexibility in where the test could occur in the clinic.

#### 2.3.5. Back Scratch Test

Participants were instructed to reach one hand over their shoulder and the other arm up the middle of their back, bringing their hands as close together as possible. Then, the distance between the extended middle fingers was measured and recorded in inches. The test was executed with both arms [32].

### 2.4. HRQoL Assessment

The Functional Assessment of Cancer Therapy‐Breast (FACT‐B) Quality of Life Version 4 questionnaire was used to evaluate HRQoL. This questionnaire consists of 37 questions across the following HRQoL domains: physical well‐being (7 items), social/family well‐being (7 items), emotional well‐being (6 items), functional well‐being (7 items), and additional concerns specific to breast cancer (10 items) [33]. Each question required a response using a 5‐point Likert scale. Higher scores indicate greater HRQoL.

### 2.5. Exercise Engagement Assessments

#### 2.5.1. Godin Analysis

To test whether the CORE clinical workflow algorithm improves self‐reported exercise engagement, all participants completed the modified Godin questionnaire [34]. This brief (four‐question) and validated questionnaire inquired about typical weekly engagement in aerobic exercise of varying intensities (i.e., strenuous, moderate, and mild) and strength training over the past month. Exercise volume was recorded as average times per week (frequency) and minutes per session (duration in minutes). This enabled the calculation of total metabolic equivalent hours per week (MET‐hr/wk), accounting for both aerobic exercise and resistance training, utilizing the following formula:

Godin=6×strenuous+3×moderate+1.5×mild+3.5×strength60 min/h.

The category (strenuous, moderate, mild, or strength training) was calculated as follows:

category=#times per week in category×#of minutes each time.

#### 2.5.2. Accelerometer Analysis

To objectively measure exercise engagement throughout the 24‐week study period, all participants were provided an ActiGraph GT9X Link (ActiGraph LLC, Pensacola, Florida, United States) triaxial accelerometer at the three aforementioned time points and instructed to wear the device on their nondominant wrist. Participants were also provided a self‐addressed, stamped envelope and instructed to mail back the device after 7 days of consecutive wear. Days were considered evaluable if the accelerometer log recorded at least 8 h of wear time, and every participant needed at least three evaluable days per time point to be included in the accelerometer analysis. Objective exercise engagement was measured as the average time (minutes) spent in moderate‐to‐vigorous physical activity (MVPA). MVPA minutes were assessed within the ActiLife software via established cut‐points reported by Freedson et al. [35], which report activity intensity in counts per minute.

### 2.6. Statistical Analysis

Descriptive statistics were reported as medians with interquartile ranges (IQRs) for continuous variables and frequencies and percentages for categorical variables. The primary analyses focused on changes in outcomes from the initial surgical consultation to the 24‐week postoperative visit. Changes in outcomes from the initial surgical consultation to the postoperative visit were secondary analyses. All data analyses were performed in SAS Version 8.3 (SAS Institute, Inc., Cary, North Carolina, United States).

#### 2.6.1. Physical Function and HRQoL

To assess changes in self‐reported physical function, a constrained linear mixed‐effects model was used with the PROMIS *T*‐score as the outcome and the group (CORE vs. SOC) as the primary predictor of interest. Covariates for this analysis were age, cancer stage, surgical oncologist, and adherence to PA guidelines. To adjust for baseline PROMIS *T*‐scores at the initial surgical consultation, both CORE and SOC group means were constrained to be equal at baseline following the methodology by Fitzmaurice et al. [36, 37]. Differences in changes in PROMIS *T*‐scores between the initial surgical consultation and the 24‐week postoperative visit, as well as between the initial surgical consultation and the postoperative visit, were reported as estimated mean differences between CORE and SOC, calculated with contrasts. Changes in all other physical function outcomes (QuickDASH, five‐time chair stand test, 10‐m walk test, and back scratch test), as well as HRQoL (FACT‐B score) from the initial surgical consultation to the 24‐week postoperative visit and the postoperative visit, were analyzed and reported in the same manner as described above.

#### 2.6.2. Exercise Engagement

To evaluate the influence of the CORE clinical workflow algorithm on changes in exercise engagement, total MET‐hr/wk as calculated from the Godin questionnaire was treated as the primary statistical endpoint over the accelerometer MVPA minutes. Total MET‐hr/wk (aerobic + strength training exercise) from the Godin questionnaire was analyzed using a constrained linear mixed‐effects model with total MET‐hr/wk as the outcome and the group (CORE vs. SOC) as the primary predictor of interest. For this analysis, the same parameters used in the PROMIS physical function statistical analysis were applied, with the exception that the PROMIS *T*‐score was included as a covariate in place of the surgical oncologist. Baseline values were similarly constrained [36, 37], and changes in MET‐hr/wk were reported as estimated mean differences, using the same approach and time points described above.

The Godin questionnaire was selected instead of accelerometry as the primary statistical endpoint to minimize potential selection bias that could have occurred when evaluating accelerometer data based on prespecified wear time (i.e., our definition of evaluable accelerometer data). For logistical purposes of trial execution, to minimize the number of on‐site participant visits (the trial opened for enrollment during the COVID‐19 pandemic) and to align the trial with routine clinical workflow, accelerometers were provided at each of the three aforementioned routine cancer care visits. This means that at the initial surgical consultation (i.e., baseline), after participants were informed of the group to which they were randomized, they were provided the accelerometer to wear for 1 week, which could have created bias as participants in CORE may have already started the intervention if they were triaged to a service pathway and able to complete their appointment during this 1‐week period.

Each participant’s daily total MVPA minutes were averaged across evaluable days. To detect differences in total average MVPA minutes between CORE and SOC at the 24‐week postoperative time point, a linear regression model was used to estimate the unadjusted treatment effect (mean difference between groups) and was adjusted for baseline measures of age, breast cancer stage, surgical oncologist, adherence to national PA guidelines, and physical function (PROMIS *T*‐score) in two‐tailed tests at an alpha of 0.05. As an exploratory analysis, the median change in MVPA minutes from the initial surgical consultation to the 24‐week postoperative visit was measured in both groups, and the difference between the two groups was estimated with a bootstrapped 95% confidence interval (CI), as the initial surgical consultation (i.e., baseline) accelerometer data were collected after open‐label treatment assignment. This analysis was 81% powered to detect an increase in CORE with an effect size of 0.5 if 80% of participants had at least three evaluable accelerometer days at the initial surgical consultation and 24‐week postoperative time points and if the covariates explained 50% of the endpoint variability. Missing accelerometer data were imputed via multiple imputation, using the mice package in R (R Core Team 2025, Vienna, Austria).

## 3. Results

### 3.1. Participant Characteristics

A total of 72 patients were enrolled and allocated to CORE or SOC in the CORE trial and are described elsewhere [26]. A total of 11 participants were no longer eligible after screening, and two did not complete the questionnaires and physical assessments at the initial surgical consultation (i.e., baseline) relevant to this investigation, leaving 59 participants for the present investigation, characterized in Table 1. At the initial surgical consultation, participants were on average 58 years of age with an overweight body mass index (BMI), primarily White and non‐Hispanic, with Stage I breast cancer. The majority of patients received radiation and hormonal therapies. While there were no differences between CORE and SOC in patient characteristics, the CORE participants showed a median PROMIS *T*‐score of 49 (IQR: 43–60), and SOC displayed a median PROMIS *T*‐score of 60 (IQR: 47–60). At the initial surgical consultation, 17% of participants met national PA guidelines based on the Godin questionnaire responses.

**Table 1:** Summary of baseline participant characteristics.

Characteristics	CORE *n* = 38	SOC *n* = 21	Total *n* = 59
	Median (1st quartile–3rd quartile)	Median (1st quartile–3rd quartile)	Median (1st quartile–3rd quartile)
Age (years)	63 (50–71)	51 (49–60)	58 (49–69)
Height (cm)	165.1 (162.6–170.2)	165.1 (162.6–165.1)	165.1 (162.6–170.2)
Body mass (kg)	70.3 (66.7–84.8)	71.2 (65.8–80.7)	70.8 (66.2–84.8)
BMI (kg/m^2^)	25.7 (23.0–30.3)	26.0 (24.5–29.6)	26.0 (23.3–30.1)
PROMIS *T*‐score	49 (43–60)	60 (47–60)	51 (44–60)
	**n (%)**	**n (%)**	**n (%)**

Race			

White	28 (74)	16 (76)	44 (75)
Unknown/not reported	10 (26)	5 (24)	15 (25)

Ethnicity			

Hispanic	1 (2.6)	0 (0)	1 (1.7)
Non‐Hispanic	33 (87)	20 (95)	53 (90)
Unknown/not reported	4 (11)	1 (4.8)	5 (8.5)

Cancer stage (*n* = 59)			

I	32 (84)	17 (81)	48 (83)
> I	1 (2.6)	4 (19)	5 (8.5)
Unknown	5 (13)	0 (0)	5 (8.5)

Postsurgery treatment type			

Adjuvant chemotherapy	6 (17)	5 (24)	11 (20)
Radiation	22 (63)	12 (57)	34 (61)
Hormone therapy	32 (91)	18 (86)	50 (89)
Immunotherapy	1 (2.9)	0 (0)	1 (1.8)

Number of treatments postsurgery			

Unimodal	10 (29)	6 (29)	16 (29)
Bimodal	18 (51)	10 (48)	28 (50)
Multimodal	5 (14)	3 (14)	8 (14)

Surgical oncologist			

Brownson	10 (27)	4 (20)	14 (25)
Matsen	11 (30)	6 (30)	17 (30)
Porretta	2 (5.4)	1 (5.0)	3 (5.3)
Rosenthal	14 (38)	9 (45)	23 (40)
Missing	1	1	2
Adherence to PA guidelines	7 (18)	3 (14)	10 (17)

*Note:* Numerical values represent the median (interquartile range) of the participant population within each group. Baseline, initial surgical consultation.

Abbreviations: CORE, Comprehensive Oncology Rehabilitation and Exercise; PA, physical activity; SOC, standard of care.

#### 3.1.1. Participant Characteristics in the Accelerometer Analysis

A subset of 38 participants had evaluable accelerometer data for all three time points, and they were included in the analysis of accelerometer data (Table 2). At the initial surgical consultation, 24% of participants met national PA guidelines based on the Godin questionnaire results in this analysis cohort. At the initial surgical consultation, there were no statistically significant differences in total MVPA minutes between CORE and SOC. Time spent in total MVPA, light‐ and moderate‐intensity activity, and percentage of time spent in moderate‐intensity activity displayed large standardized mean differences (SMDs) of greater than 0.4, indicating that the two groups were different at the initial surgical consultation time point. The SOC group displayed 145 ± 47 total MVPA minutes, whereas the CORE group presented with 117 ± 72 total MVPA minutes at the initial surgical consultation (95% CI −0.09 to 1.0, SMD 0.47, *p* = 0.07; Table S1 in the Supporting Information section).

**Table 2:** Summary of baseline participant characteristics of the accelerometer analysis cohort.

Characteristics	CORE *n* = 24	SOC *n* = 14	Total *n* = 38
	Median (1st quartile–3rd quartile)	Median (1st quartile–3rd quartile)	Median (1st quartile–3rd quartile)
Age (years)	59 (50–67)	56 (50–69)	58 (50–67)
Height (cm)	166.4 (162.6–170.2)	165.1 (162.6–172.1)	165.1 (162.6–165.1)
Body mass (kg)	68.5 (66.7–75.7)	76.7 (68.0–93.0)	70.8 (67.1–80.7)
BMI (kg/m^2^)	24.7 (22.3–28.3)	28.8 (25.1–34.1)	25.7 (22.5–29.6)
Baseline PROMIS *T*‐score	56.5 (43.2–60.1)	51.8 (45.4–60.1)	53.0 (43.6–60.1)
	**n (%)**	**n (%)**	**n (%)**

Race			

White	19 (79)	11 (79)	30 (79)
Unknown/not reported	5 (21)	3 (21)	8 (21)

Ethnicity			

Hispanic	0 (0)	0 (0)	0 (0)
Non‐Hispanic	20 (83)	14 (100)	34 (89)
Unknown/not reported	4 (17)	0 (0)	4 (11)

Cancer stage			

I	22 (92)	12 (86)	34 (89)
> I	0 (0)	2 (14)	2 (5.3)
Unknown	2 (8.3)	0 (0)	2 (5.3)

Postsurgery treatment type			

Adjuvant chemotherapy	3 (14)	4 (29)	7 (19)
Radiation	13 (59)	9 (64)	22 (61)
Hormone therapy	21 (95)	12 (86)	33 (92)
Immunotherapy	1 (24.5)	0 (0)	1 (2.8)

Number of treatments postsurgery			

Unimodal	7 (32)	3 (21)	10 (28)
Bimodal	11 (50)	8 (57)	19 (53)
Multimodal	3 (14)	2 (14)	5 (14)

Surgical oncologist			

Brownson	3 (23)	5 (22)	8 (22)
Matsen	7 (30)	5 (38)	12 (33)
Porretta	2 (8.7)	0 (0)	2 (5.6)
Rosenthal	9 (39)	5 (38)	14 (39)
Missing	1	1	2
Adherence to PA guidelines	6 (25)	3 (21)	9 (24)

*Note:* Numerical values represent the median (interquartile range) of the participant population within each group. Baseline, initial surgical consultation.

Abbreviations: CORE, Comprehensive Oncology Rehabilitation and Exercise; PA, physical activity; SOC, standard of care.

### 3.2. Physical Function Assessments

#### 3.2.1. PROMIS Analysis

**Figure 1:**
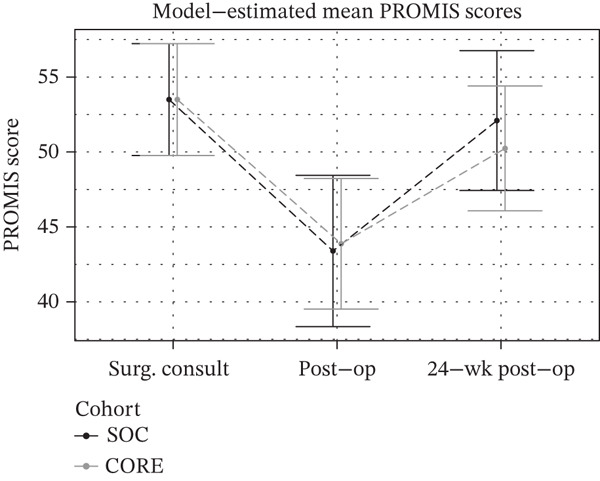
PROMIS *T*‐scores at each time point by group. Abbreviations: 24‐wk post‐op, 24‐week postoperative visit (SOC: *n* = 18; CORE: *n* = 33); CORE, Comprehensive Oncology Rehabilitation and Exercise; post‐op, postoperative visit (SOC: *n* = 19; CORE: *n* = 35); SOC, standard of care; surg. consult, initial surgical consultation (SOC: *n* = 21; CORE: *n* = 38).

A statistically significant difference in PROMIS *T*‐score change between groups (CORE vs. SOC) from the initial surgical consultation to the 24‐week postoperative visit was not observed (mean difference: −1.86, 95% CI −6.02 to 2.3; effect size: −0.22, 95% CI −0.71 to 0.27, *p* = 0.37). For the change from the initial surgical consultation to the postoperative visit, a mean difference of 0.48 (95% CI −4.71 to 5.67) and an effect size of 0.06 (95% CI −0.56 to 0.67, *p* = 0.85) were observed favoring CORE. Mean PROMIS *T*‐scores at each time point by group are presented in Figure 1.

#### 3.2.2. Other Physical Function Measures

The difference in change scores between CORE and SOC from the initial surgical consultation (i.e., baseline) to the 24‐week postoperative visit and from the initial surgical consultation to the postoperative visit is displayed in Table 3. Findings from the 10‐m walk test revealed a small to moderate benefit in the CORE group from the initial surgical consultation to the 24‐week postoperative visit (estimated mean change: 0.64, 95% CI −0.25 to 1.53; effect size: 0.32, 95% CI −0.13 to 0.77, *p* = 0.16). Statistically significant differences in other physical function outcomes were not observed.

**Table 3:** Difference in change scores for physical function outcomes.

Outcome	Difference in change scores (CORE–SOC)	Estimate (95% CI)	Effect size (95% CI)	*p* value
QuickDASH	Baseline to 24‐wk post‐op	1.94 (−6.36 to 10.24)	0.21 (−0.67 to 1.08)	0.65
QuickDASH	Baseline to post‐op	−6.62 (−19.08 to 5.84)	−0.7 (−2.02 to 0.62)	0.29
Five‐time chair stand test	Baseline to 24‐wk post‐op	0.31 (−1.76 to 2.37)	0.07 (−0.38 to 0.51)	0.77
Five‐time chair stand test	Baseline to post‐op	−0.56 (−2.61 to 1.5)	−0.12 (−0.56 to 0.32)	0.59
Ten‐meter walk test	Baseline to 24‐wk post‐op	0.64 (−0.25 to 1.53)	0.32 (−0.13 to 0.77)	0.16
Ten‐meter walk test	Baseline to post‐op	0.04 (−1.26 to 1.34)	0.02 (−0.63 to 0.67)	0.95
Right shoulder mobility	Baseline to 24‐wk post‐op	0.84 (−0.59 to 2.27)	0.19 (−0.13 to 0.51)	0.24
Right shoulder mobility	Baseline to post‐op	−0.78 (−2.27 to 0.7)	−0.18 (−0.51 to 0.16)	0.29
Left shoulder mobility	Baseline to 24‐wk post‐op	0.14 (−1.53 to 1.81)	0.03 (−0.36 to 0.42)	0.86
Left shoulder mobility	Baseline to post‐op	−0.17 (−2.05 to 1.7)	−0.04 (−0.48 to 0.4)	0.85

*Note:* The QuickDASH score was reported on a 0–100 scale; the five‐time chair stand test and the 10‐m walk test were measured in seconds; shoulder mobility was measured via the back scratch test in inches. Baseline, initial surgical consultation.

Abbreviations: CI, confidence interval; CORE, Comprehensive Oncology Rehabilitation and Exercise; QuickDASH, Quick Disabilities of the Arm, Shoulder, and Hand; QoL, quality of life; SOC, standard of care.

### 3.3. Quality of Life Assessment

**Figure 2:**
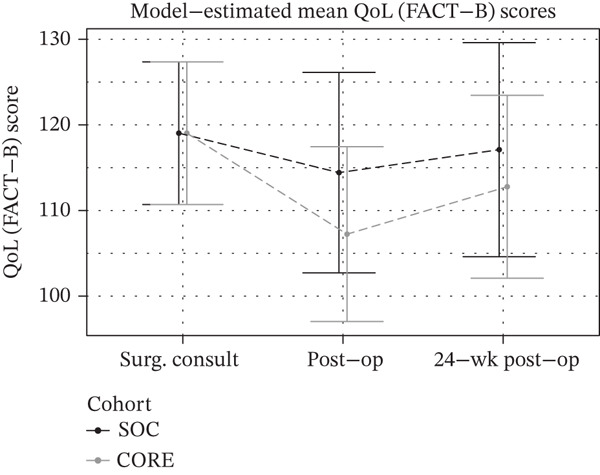
FACT‐B scores at each time point by group. Abbreviations: 24‐wk post‐op, 24‐week postoperative visit (SOC: *n* = 18; CORE: *n* = 33); CORE, Comprehensive Oncology Rehabilitation and Exercise; FACT‐B, Functional Assessment of Cancer Therapy‐Breast; post‐op, postoperative visit (SOC: *n* = 19; CORE: *n* = 35); SOC, standard of care; surg. consult, initial surgical consultation (SOC: *n* = 21; CORE: *n* = 38).

A statistically significant difference between groups was not observed for HRQoL. The mean differences in change in FACT‐B scores between groups from the initial surgical consultation to the 24‐week postoperative visit and to the postoperative visit were −4.33 (95% CI −16.54 to 7.89; effect size: −0.3, 95% CI −1.15 to 0.55,*p* = 0.48) and −7.19 (95% CI −17.97 to 3.6; effect size: −0.5, 95% CI −1.25 to 0.25,*p* = 0.19), respectively. The model‐estimated mean FACT‐B scores over time are displayed in Figure 2.

### 3.4. Exercise Engagement Assessments

#### 3.4.1. Godin Analysis

**Figure 3:**
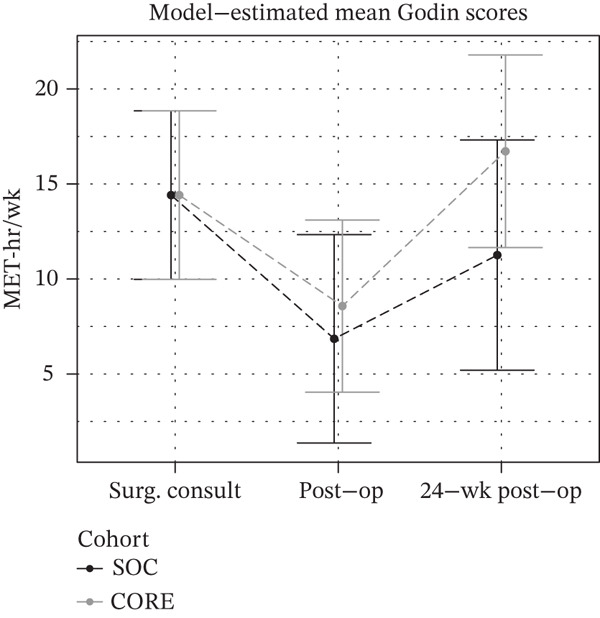
Godin total MET‐hr/wk at each time point by group. Abbreviations: 24‐wk post‐op, 24‐week postoperative visit (SOC: *n* = 16; CORE: *n* = 33); CORE, Comprehensive Oncology Rehabilitation and Exercise; post‐op, postoperative visit (SOC: *n* = 17; CORE: *n* = 35); SOC, standard of care; surg. consult, initial surgical consultation (SOC: *n* = 20; CORE: *n* = 38); total MET‐hr/wk, total metabolic equivalent hours per week (accounting for engagement in both aerobic exercise and resistance training).

The CORE intervention resulted in a modest advantage compared with SOC, with a mean difference in Godin total MET‐hr/week change from the initial surgical consultation to the 24‐week postoperative visit of 5.46 (95% CI −1.06 to 11.98) and an effect size of 0.36 (95% CI −0.07 to 0.78,*p* = 0.10). For the secondary analysis (difference in change from the initial surgical consultation to the postoperative visit), the CORE group displayed marginally increased Godin total MET‐hr/week compared with SOC (mean difference: 1.72, 95% CI −3.97 to 7.41; effect size: 0.11, 95% CI −0.26 to 0.48, *p* = 0.55). The model‐estimated mean Godin total MET‐hr/week at each time point by group is displayed in Figure 3.

#### 3.4.2. Accelerometer Analysis

Exercise engagement was assessed as the average total time spent in MVPA in minutes. At the 24‐week postoperative point, the average MVPA minutes were modestly lower among participants in CORE compared with SOC, with an adjusted mean difference of −12 min (95% CI −57 to 33, *p* = 0.6).

**Figure 4:**
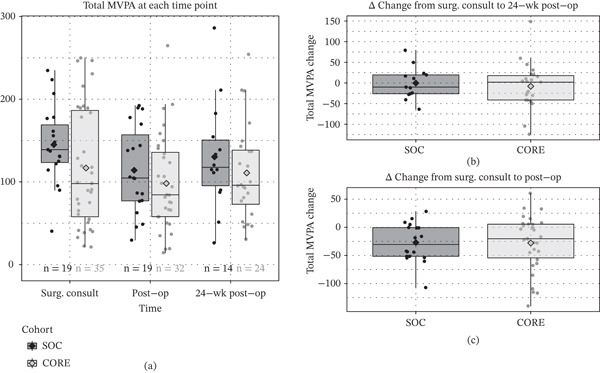
Accelerometer results at each time point by group. Abbreviations: 24‐wk post‐op, 24‐week postoperative visit; CORE, Comprehensive Oncology Rehabilitation and Exercise; MVPA, moderate‐to‐vigorous physical activity; post‐op, postoperative visit; SOC, standard of care; surg. consult, initial surgical consultation. (a) Total MVPA minutes at each time point. (b) Delta (*Δ*) change in total MVPA minutes from surg. consult to 24‐wk post‐op. (c) Delta (*Δ*) change in total MVPA minutes from surg. consult to post‐op. Diamonds indicate the mean in each group.

In our exploratory analysis evaluating delta change in MVPA from the initial surgical consultation to the 24‐week postoperative visit, the average total MVPA minutes increased by a median of 2 min within CORE, while SOC decreased by a median of 10 min (difference of medians: 12 min, bootstrapped 95% CI −37 to 40). Participant demographics of patients who improved and did not improve total MVPA minutes from the initial surgical consultation to the 24‐week postoperative visit were similar (Table S2 in the Supporting Information section). However, compared with participants who did not improve average total MVPA minutes (60.1, IQR: 46.5–60.1), participants who did improve average total MVPA minutes had a PROMIS *T*‐score of 49.4 (IQR: 42.5–60.1) at the initial surgical consultation visit. The total MVPA at each time point by group and the delta change between time points are depicted in Figure 4.

## 4. Discussion

In this investigation, we aimed to evaluate the preliminary effectiveness of our CORE clinical workflow algorithm on physical function, HRQoL, and exercise engagement compared with SOC. While patients receiving the CORE algorithm as part of routine breast cancer care appeared to have increased exercise engagement and gait speed (a measure of physical function) 24 weeks after surgery, statistically significant differences between groups in all outcomes examined were not observed, and CIs for most outcomes were wide. Together, these findings are promising yet demonstrate uncertainty in determining whether CORE is beneficial in changing these outcomes.

Although the observed improvement in exercise engagement among CORE participants was not statistically significant, it is noteworthy to point out that the SOC group displayed higher baseline exercise engagement (145 ± 47 total MVPA minutes) compared with CORE (117 ± 72 total MVPA minutes). For logistical purposes to ensure successful execution of the triaging tool within the algorithm at routine clinic visits, consent, enrollment, and randomization occurred before women checked in for the initial surgical consultation clinic visit (i.e., baseline study day). This resulted in the accelerometer wear occurring after revelation of study arm assignment, which may have influenced exercise engagement for individuals in the SOC arm throughout the remainder of the trial. Unrelated but also worth mentioning, while not achieving statistical significance, participants randomly assigned to CORE exhibited a worse functional status at baseline compared with SOC; specifically, the PROMIS *T*‐score was 49 in CORE, indicating a mild functional deficit [38], compared with a PROMIS *T*‐score of 60 in SOC. Baseline functional status may have influenced changes in outcomes over time in this study.

The CORE pilot trial was informative in demonstrating proof of trial feasibility and acceptability among patients, providers, and their clinic teams [26]. We observed overwhelmingly positive qualitative data supporting the utility of the CORE clinical workflow algorithm to improve physical function, independence, and quality of life throughout breast cancer care [26]. One hundred percent of feedback provided in the focus group relative to the influence of the CORE clinical workflow algorithm on algorithm outcomes (i.e., reduced symptoms, activity, function, plans for exercise, and exercise needs) evaluated was positive [26]. For example, one participant reported that her mobility and strength were better after her cancer diagnosis compared with before, which she attributed to the CORE algorithm being part of routine care [26]. Many reported the attenuation of side effects and the ability to return to activities they enjoyed before their cancer diagnosis, such as skiing [26]. Our rich qualitative data support the advantage of the CORE clinical workflow algorithm to positively impact physical function, HRQoL, and exercise engagement, warranting further investigation with a larger, adequately powered trial to interrogate effectiveness.

Furthermore, the CORE pilot trial informed how to effectively align collection of outcome measures with routine clinical workflow in preparation for a future, adequately powered trial. This information is critical and of high utility in efforts to facilitate more exercise oncology clinical trials that implement exercise and rehabilitation services into routine cancer care. To our knowledge, there are only two studies in the United States that have aimed to integrate exercise as part of routine cancer care [39, 40]. One study is a quality improvement project evaluating the implementation of the ACSM’s Exercise in Cancer Evaluation Decision Support (EXCEEDS) algorithm within chemotherapy infusion clinics [39], and the other is a clinical trial embedding physical therapists within the lung surgical oncology clinics, the Precision‐Exercise‐Prescription (PEP) trial [40]. These studies represent two different and needed areas of research within the field of exercise oncology. The EXCEEDS study included referral to both exercise and rehabilitation services and evaluated implementation among a heterogeneous sample of patients with cancer undergoing active treatment [39]. This work supports our findings in that integrating an exercise and rehabilitation referral system within clinical workflow is feasible. A strength of this EXCEEDS quality improvement project is the heterogeneity of cancer sites included in the sample. Our work complements these findings and collectively exhibits the feasibility of clinical workflow algorithms for exercise and rehabilitation referrals among varying cancer types and stages during active cancer treatment. On the other hand, the PEP trial embedded rehabilitation professionals in lung surgical oncology clinics, eliminating the need for referrals [40]. Effectiveness of the PEP model was evaluated in this Phase III, adequately powered randomized controlled trial and revealed statistically significant improvements in physical function and fatigue [40]. This work demonstrates the effectiveness of integrating exercise and rehabilitation as part of routine cancer care in the context of surgical oncology for lung cancer and aligns with our preliminary findings, which reveal the possible utility of CORE in breast surgical oncology. Broadly, our work along with the work of others supports recent guidelines and recommendations established by the ACSM and ASCO supporting exercise across the cancer care continuum and aligns with ACSM policy efforts to get exercise services during cancer care reimbursed by third‐party payers.

The present investigation has many strengths, such as evaluating a feasible and acceptable (among both patients and clinic staff) triaging tool that is short (minimizing time burden for patients and clinic staff) and effective at directing patients to the right service (i.e., exercise or rehabilitation) at the right time throughout care, inclusion of objective and subjective outcome measures, and alignment of algorithm and outcome assessment execution with routine breast cancer care. However, our investigation is not without limitations, such as a small sample size inhibiting adequate power to detect statistically significant differences between groups for outcomes. The 2:1 randomization allocation enabled adequate power to evaluate feasibility and acceptability (primary aims) but limits our ability to assess preliminary effectiveness and draw conclusions from group comparisons. As noted previously, randomization and group revelation before the baseline study day may have influenced some outcomes. Further, our sample was homogeneous pertaining to race, ethnicity, and breast cancer stage, yet adjuvant treatment type varied, supporting the generalizability of these findings in the sense of feasibility and acceptability.

Future research should include a larger sample size, a more diverse sample, and inclusion of patients with breast cancer and all treatment plans at the time of diagnosis, as opposed to limiting it to surgery as first‐line treatment, which results in earlier‐stage breast cancer. Future research may also test the CORE clinical workflow algorithm in other cancer types to build the evidence base in cancer sites that are less represented in the exercise oncology literature (i.e., cancer sites other than breast, prostate, lung, and colorectal). Overall, the CORE clinical workflow algorithm demonstrates promise as a tool to connect individuals with cancer with the right care at the right time from diagnosis and beyond to improve survivorship outcomes.

## Funding

This study was funded by 5 For The Fight, a Qualtrics Inc.–sponsored nonprofit organization.

## Ethics Statement

This study was performed in line with the principles of the Declaration of Helsinki. Approval was granted by the Ethics Committee of the University of Utah (IRB #00137018).

## Conflicts of Interest

The authors declare no conflicts of interest.

## Supporting information


**Supporting Information 1.** Additional supporting information can be found online in the Supporting Information section. Supporting Information. This supporting information provides additional information and data related to the study presented in the main manuscript, and it is essential for further describing the physical activity data of the participants in the trial and contributing to the interpretation of the results. Table S1: Descriptive data related to differences in objective exercise engagement at baseline. Table S2: Summary of participant characteristics for patients who did and did not increase total MVPA.

## Data Availability

The data that support the findings of this study are available from the corresponding author upon reasonable request.
