# Supplementary material for: Preliminary Effectiveness of the Comprehensive Oncology Rehabilitation and Exercise (CORE) Clinical Workflow Algorithm on Health Outcomes During Nonmetastatic Breast Cancer Care
Source: Int J Breast Cancer. 2026 Jun 11;2026:8894250. doi: 10.1155/ijbc/8894250 (PMC13255010; doi:10.1155/ijbc/8894250)
Supplement: Supplementary file 1 — Supporting Information 1. Additional supporting information can be found online in the Supporting Information section. Supporting Information. This supporting information provides additional information and data related to the study presented in the main manuscript, and it is essential for further describing the physical activity data of the participants in the trial and contributing to the interpretation of the results. Table S1: Descriptive data related to differences in objective exercise engagement at baseline. Table S2: Summary of participant characteristics for patients who did and did not increase total MVPA. [file IJBC-2026-8894250-s001.docx]

Supplementary Material

Table S1. Differences in Baseline Objective Exercise Engagement

| Characteristics | CORE  n=35^1^ | SOC  n=19^1^ | Total  n=54^1^ | SMD | 95% CI | p-value |
| --- | --- | --- | --- | --- | --- | --- |
| Total MVPA | 117 (72) | 145 (47) | 127 (65) | 0.47 | -0.09, 1.0 | 0.07 |
| Percentage in MVPA | 12 (7) | 15 (6) | 13 (7) | 0.45 | -0.12, 1.0 | 0.10 |
| Light | 456 (64) | 463 (80) | 458 (69) | 0.10 | -0.46, 0.65 | 0.7 |
| Moderate | 117 (72) | 145 (47) | 127 (65) | 0.47 | -0.09, 1.0 | 0.07 |
| Percent in Light | 49 (10) | 47 (9) | 48 (9) | -0.20 | -0.76, 0.36 | 0.7 |
| Percent in Moderate | 12 (7) | 15 (6) | 13 (7) | 0.45 | -0.12, 1.0 | 0.10 |
| Step Count | 9,572 (3,232) | 10,778 (2,530) | 9,996 (3,035) | 0.42 | -0.14, 0.99 | 0.2 |

*MVPA,* Moderate-Vigorous Physical Activity Minutes*; Baseline*, Initial Surgical Consultation; *CORE*, Comprehensive Oncology Rehabilitation and Exercise; *SOC,* Standard of Care; *CI*, Confidence Interval; *SMD*, Standardized Mean Difference. Numerical values represent the mean (standard deviation) of the participant population within each group. The p-value was calculated via Wilcoxon rank sum exact test.

Table S2. Summary of Participant Characteristics for Patients who did and did not increase Total MVPA

| Characteristics | Did not improve  n=18 | Improved  n=18 | Missing data  n=2 |
| --- | --- | --- | --- |
|  | Median  (1^st^,3^rd^ Quartile) | Median  (1^st^,3^rd^ Quartile) | Median  (1^st^,3^rd^ Quartile) |
| Age (years) | 56 (51, 67) | 59 (50, 74) | 59 (45, 72) |
| Height (cm) | 165.1 (162.6, 170.2) | 165.1 (162.6, 170.2) | 165.1, (165.1, 165.1) |
| Body Mass (kg) | 69.4 (66.2, 78.5) | 72.1 (68.0, 93.4) | 73.9 (54.9, 93.0) |
| BMI (kg/m^2^) | 25.8 (22.5, 28.8) | 25.7 (22.8, 34.3) | 27.1 (20.1, 34.1) |
| Baseline PROMIS T-score | 60.1 (46.4, 60.1) | 49.4 (42.5, 60.1) | 55.3 (50.5, 60.1) |
|  | n (%) | n (%) | n (%) |
| Race |  |  |  |
| White | 13 (72) | 15 (83) | 2 (100) |
| Unknown/Not Reported | 5 (28) | 3 (17) | 0 (0) |
| Ethnicity |  |  |  |
| Non-Hispanic | 16 (89) | 16 (89) | 2 (100) |
| Unknown/Not Reported | 2 (11) | 2 (11) | 0 (0) |
| Group |  |  |  |
| SOC | 7 (39) | 6 (33) | 1 (50) |
| CORE | 11 (61) | 12 (67) | 1 (50) |
| Cancer Stage |  |  |  |
| I | 16 (89) | 16 (89) | 2 (100) |
| >I | 1 (5.6) | 1 (5.6) | 0 (0) |
| Unknown | 1 (5.6) | 1 (5.6) | 0 (0) |
| Post-Surgery Treatment Type |  |  |  |
| Adjuvant Chemotherapy | 4 (24) | 3 (18) | 0 (0) |
| Radiation | 11 (65) | 10 (59) | 1 (50) |
| Hormone Therapy | 15 (88) | 17 (100) | 1 (50) |
| Immunotherapy | 1 (5.9) | 0 (0) | 0 (0) |
| Number of Treatments Post-Surgery |  |  |  |
| Unimodal | 5 (29) | 5 (29) | 0 (0) |
| Bimodal | 7 (41) | 11 (65) | 1 (50) |
| Multimodal | 4 (24) | 1 (5.9) | 0 (0) |
| Surgical Oncologist |  |  |  |
| Brownson | 2 (12) | 4 (24) | 2 (100) |
| Matsen | 5 (29) | 7 (41) | 0 (0) |
| Porretta | 2 (12) | 0 (0) | 0 (0) |
| Rosenthal | 8 (47) | 6 (35) | 0 (0) |
| Missing | 1 | 1 | 0 |
| Adherence to PA Guidelines | 4 (22) | 4 (22) | 1 (50) |

*MVPA,* Moderate-Vigorous Physical Activity Minutes*; CORE*, Comprehensive Oncology Rehabilitation and Exercise; *SOC,* Standard of Care; *PA*, Physical Activity; *Baseline*, Initial Surgical Consultation. Numerical values represent the median (interquartile range) of the participant population within each group.
